# Our Children’s Teachers

**Published:** 1878-06

**Authors:** 


### OUR CHILDREN'S TEACHERS.

A seventy-five cent volume called "Uses and Abuses of Air," has never yielded its talented author a dollar's profit, and yet it is one of the most practically useful books on that subject which we have seen. But it is not the taste of the times to patronize the books, papers, magazines, which are best calculated to promote public good, and advance the true and permanent interests of the masses. This is the reign of cheap literature. The great patronized now is, pictorial magazines, weeklies filled with wishey-washey love tales, or stories of the terrible, with "horrible" headings. Scarce anything attracts attention now, short of a "midnight murder," or a "shocking catastrophe," pandering to the passions in morals, or in politics, stirring up brother to fiercer fight against his brother, or stimulating prurient searches after things which ought to remain unexposed; while better books, books whose aim and end is to instruct the masses as to the preservation of health, the maintenance of good constitutions; which seek to invite the young to profitable industries, to elevating amusements, and refining associations, cumber the shelves of the philanthropic bookseller for years together.

